# The Role of Hypothalamic Neuropeptides in Neurogenesis and Neuritogenesis

**DOI:** 10.1155/2016/3276383

**Published:** 2016-01-13

**Authors:** Jan Bakos, Martina Zatkova, Zuzana Bacova, Daniela Ostatnikova

**Affiliations:** ^1^Institute of Experimental Endocrinology, Slovak Academy of Sciences, Vlarska 3, 833 06 Bratislava, Slovakia; ^2^Institute of Physiology, Faculty of Medicine, Comenius University, Sasinkova 2, 813 72 Bratislava, Slovakia; ^3^Department of Normal and Pathological Physiology, Faculty of Medicine, Slovak Medical University, Limbova 12, 833 03 Bratislava, Slovakia

## Abstract

The hypothalamus is a source of neural progenitor cells which give rise to different populations of specialized and differentiated cells during brain development. Newly formed neurons in the hypothalamus can synthesize and release various neuropeptides. Although term neuropeptide recently undergoes redefinition, small-size hypothalamic neuropeptides remain major signaling molecules mediating short- and long-term effects on brain development. They represent important factors in neurite growth and formation of neural circuits. There is evidence suggesting that the newly generated hypothalamic neurons may be involved in regulation of metabolism, energy balance, body weight, and social behavior as well. Here we review recent data on the role of hypothalamic neuropeptides in adult neurogenesis and neuritogenesis with special emphasis on the development of food intake and social behavior related brain circuits.

## 1. Introduction

During early developmental periods, rapid proliferation, differentiation, and migration of new progenitor cells occur especially in the hippocampus, subventricular zone, and olfactory bulb [1]. Recent studies suggest that newborn neural cells may be found in the hypothalamus and they produce various neuropeptides [2]. Current evidence indicates that continuous neurogenesis takes place during development of neural system and processes of generation and maturation of neurons extend to adulthood [3]. Many factors influence adult brain neurogenesis such as hormones, growth factors, and neurotransmitters [4]. Newly generated neurons form initial neurites which differentiate into long-distance projections called axons or into multiple short length dendrites. Brain connectivity and promotion of neurite outgrowth are tightly regulated by cytoskeletal components, microtubule proteins, actin-binding proteins, Rho pathway signaling proteins, synaptic scaffolding proteins, adhesion molecules, and locally secreted neuropeptide hormones [5, 6]. The role of neuropeptides in brain development has been extensively studied and thus certain neuropeptides have been already associated with neurogenesis [7]. Proper time course of generation of specific neuron populations and their interconnections are important factors in hypothalamic development. The role of neuropeptides in neurite growth and formation of neural circuits is far less clear. Many studies suggest that developmental abnormalities in specific hypothalamic circuits are associated with obesity, sleep disorders, anxiety, depression, and autism [8, 9]. Growth and guidance of neurites from and to the hypothalamus is essential for understanding their pathogeneses. The ability of neuropeptides to modulate neurogenesis and neurite growth is discussed in the present review.

## 2. Neuropeptides

Neuropeptides represent large and diverse group of molecules responsible for communication among cells in the central nervous system (CNS). Although neuropeptides may be located in the periphery and also play a role in control of peripheral functions, their major effects are within the CNS, by taking part in the regulation of thermoregulation, food and water intake, circadian rhythms, and sexual and reproductive behavior. A molecule can be considered as a neuropeptide, when it possesses distinct properties: (1) it is a small-size protein molecule, (2) it is produced and secreted by cells of the nervous system, and (3) it plays a specific role in the regulation of neuronal cells [10]. At least in mammals, neuropeptides are encoded by over 70 genes [10]. The size of the molecule is usually between 3 and 100 amino acids (AC), while more than 75% of known neuropeptides have a molecule of less than 30 AC [11]. Neuropeptide synthesis takes place mostly in a physiologically inactive form as the pre-pro-molecules. The precursor, before being stored or released from the cell, is typically degraded to short chain of AC through endopeptidases in the Golgi apparatus or directly in the secretory vesicles [12, 13]. Nevertheless, some neuropeptide molecules undergo further posttranslational modifications, necessary to ensure their stability and full biological activity, such as phosphorylation, acetylation, sulphonation, or removal of their terminal part. Metabolic changes necessary to achieve a fully active form of the neuropeptide are sometimes so intensive that the result leads to an extreme shortening of the peptide chain. Particularly, thyrotropin releasing hormone (TRH) consists only of three amino acids, compared to its much larger pre-pro-form [14]. Neuropeptides are secreted from large dense core vesicles by regulated secretion. They may be stored in vesicles together with other low molecular weight neuropeptides or even with other neurotransmitters [15]. Their secretion is not necessarily limited to the synaptic cleft; however it usually occurs in the close vicinity [14, 16, 17]. There are also reported cases of secretion from the cell body or from dendritic spines [18]. Neuropeptides play a crucial role in cell-to-cell communication by affecting gene expression [19], synaptogenesis [20], and modulation of membrane excitability [21]. Some neuropeptides even may act as neurotransmitters [22]. Despite the often generalized physiological effects of many neuropeptides, time of their biological activity in the circulation is significantly limited. For instance, oxytocin has a half-life in blood of approximately 120 seconds, compared to the half-life in the CNS extracellular space, which is about 20 minutes [23]. Diffusion through the extracellular space and binding to membrane receptors are in a case of robust neuropeptide much slower, however, from the physical-chemical point of view, more solid [24]. Slower modulatory effect on the potential of the postsynaptic membrane is linked to the mechanism of the neuropeptide receptor pathway. Most neuropeptides have their own specific receptor coupled with G-protein. Although the size of neuropeptide molecules is relatively large compared to classical neurotransmitters, affinity to the specific receptors is approximately 1000-fold higher than that of the neurotransmitter, thus being capable of eliciting a biological response at lower concentrations [21].

## 3. Classification of Neuropeptides

Up to date, the different databases (NeuroPep, NeuroPedia, http://www.neuropeptides.nl/) cover over 5900 neuropeptides divided into large groups [10, 11, 25]. While the number of neuropeptides in vertebrates reaches nearly 2500 [11], it can be expected that the list is still not complete. The division into families may be based on similarities in the gene structure (e.g., calcitonin gene family, F- and Y-amide gene family), molecule structure (e.g., oxytocin/vasopressin family, insulin/insulin-like growth factor (IGF) family), function (e.g., opioid neuropeptide family, adipose neuropeptide family), or localization of neurons producing each neuropeptide (hypothalamic neuropeptide family, hypophyseal neuropeptide family). Many novel neuropeptides remain unclassified. One given peptide is often localized to different brain areas and it is involved in more than one biological function. Neuropeptides expressed in hypothalamic neurons form a large group of well-described peptides with a variety of peripheral (endocrine) and central functions.

## 4. Structure of the Hypothalamus

### 4.1. Hypothalamic Neuronal Populations

**Figure 1:**
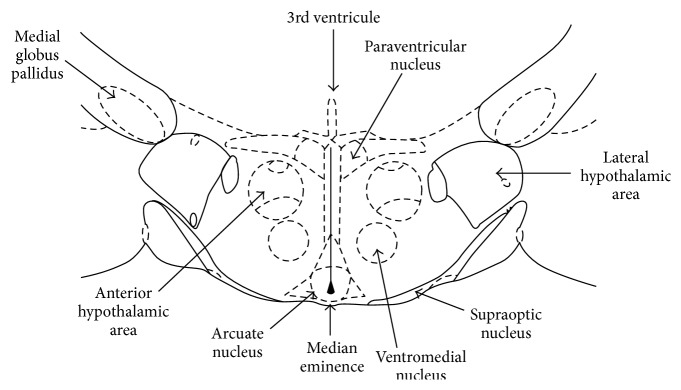
Overview of the major hypothalamic nuclei producing neuropeptides. Paraventricular and supraoptic nuclei contain neurons producing corticotrophin-releasing hormone, urocortins, thyrotropin releasing hormone, oxytocin, and vasopressin. Ventromedial nucleus neurons produce Substance P, enkephalins, and Neuropeptide Y. Arcuate nucleus neurons express agouti-related peptide, Neuropeptide Y, proopiomelanocortin, melanocyte-stimulating hormones, adrenocorticotropic hormone-stimulating hormone, and endorphins (modified according to Paxinos and Watson, 1997 [30]).

The hypothalamus is an ancient and conserved forebrain area, traditionally divided to lateral, medial, and periventricular part and furthermore to the distinct functional nuclei [26]. Hypothalamic nuclei contain diverse cell populations [27], which can be defined by specific patterns of gene expression, such as ion channels, transcription factors, and neuropeptides (Figure 1). Populations of neurons secreting various neuropeptides located in the lateral hypothalamus play a major role in food intake. In the arcuate nucleus, neurons express orexigenic agouti-related peptide (AgRP), Neuropeptide Y (NPY), and anorexigenic peptides proopiomelanocortin (POMC). Another group of neurons produce peptides promoting food intake—orexin and melanin-concentrating hormone (MCH) [28]. Nevertheless, arcuate hypothalamic neurons that produce proopiomelanocortin (POMC) secrete an anorexic neuropeptide melanocyte-stimulating hormone (*α*-MSH), a proteolytic product of POMC. Another endogenous peptide product of the POMC represents adrenocorticotropic hormone (ACTH), *β*- and *γ*-melanocyte-stimulating hormones (*β*- and *γ*-MSH), and *β*- endorphin. Located lateral to the arcuate nucleus, the ventromedial nucleus is the major constituent of the mediobasal hypothalamus. Ventromedial nucleus is important in the regulation of sexual behavior and analgesia [29]. Large amount of neuropeptides, such as Substance P, enkephalins, and NPY, is synthesized in the ventromedial nucleus [29]. The periventricular part of the hypothalamus is responsible for secretion of NPY, TRH, somatostatin, leptin, gastrin, and gonadotropin-releasing hormone. Paraventricular and supraoptic nuclei of the hypothalamus contain neurons producing corticotrophin-releasing hormone (CRH), TRH, oxytocin, and vasopressin (Figure 1).

### 4.2. Hypothalamic Neuronal Connections

The hypothalamus sends information directly to other brain areas and to periphery by neural projections and indirectly to the blood stream. Neuroendocrine regulation is mediated mostly via hypothalamic-pituitary-adrenal, hypothalamic-pituitary-thyroideal, and hypothalamic-pituitary-gonadal axes. Proper transmission of neural signals from periphery to the hypothalamus is mediated by visceral and somatosensory inputs. Furthermore, control of the autonomic nervous system is assured by direct outputs to the brain stem. It is very well documented that neural projections originating or terminating in the hypothalamus are involved in regulation of food, energy, and heat balance. The hypothalamo-neurohypophyseal system plays a fundamental role in the control of fluid and electrolyte balance forming complex neural network responsible for an integrated response [31]. It is also known that olfactory receptor neurons form circuits with hypothalamic subregions [32]. Next, pathways from retina to the suprachiasmatic nucleus of the hypothalamus are involved in regulation of circadian rhythms and light-dark cycle. Projections from the hypothalamus to the cerebral cortex participate in the control of sexual, reproductive, and social behavior [26, 27, 33].

## 5. Development of the Hypothalamus

The relevant information on functional organization of intra- and interhypothalamic circuits has dramatically increased in the last decades. The hypothalamus has complex connections with other brain regions ranging from retina to cortex. These connections are formed during embryonic development; however they are further rearranged later in life under conditions of nutritional state, stress, or lactation [50–52]. Hypothalamic circuits, connections, and pathways are thus dynamically regulated resulting in marked changes of brain plasticity manifested by enhanced neurogenesis and neuritogenesis. It is known that hypothalamic neurogenic niche (hypothalamic proliferating zone) lining the ventral portion of the third ventricle consists of cells with high proliferative activity even in the adult age [53, 54]. Precursor cells lining the third ventricle are able to receive diverse molecular signals, for example, neuropeptides, and growth factors present in the cerebrospinal fluid. Mounting evidence suggest that hypothalamic neurogenic capacities can be affected in the adult mammalian brain [55]. In addition to production of neurons, shift from neurogenesis to gliogenesis has been shown in the developing hypothalamus [56]. Traditionally, it was believed that most of the hypothalamus is formed in three neurogenetic stages producing neurons that progressively accumulate; however recent studies suggest that hypothalamic progenitor cells have common origin [56]. Nevertheless, it is known that the hypothalamus develops from the rostral diencephalon and cells from various origins migrate to the hypothalamic region during development. Hypothalamic neuron populations are under the control of many intracellular transcriptional factors. The most known are sonic hedgehog protein (Shh) [56, 57] and a group of proteins belonging to wingless family (Wnt), which has been long known to be involved in patterning during development [54]. Shh is considered as a morphogen that regulates the dorsoventral patterning of central nervous system. Recent study has demonstrated that Shh coordinates anteroposterior and dorsoventral patterning in the hypothalamus [58]; moreover it has been reported that chemorepulsive effect of Shh repels hypothalamic axons from the ventricular zone of the hypothalamus and results in their growth in fascicules [59]. Differentiated neurons or glia cells cease to express Shh [60]. Wnt signaling is required for neurogenesis and eventually for anterior patterning, including the region that gives rise to the hypothalamus [54, 61]. Newborn cells have been described in the adult hypothalamus, suggesting constitutive neurogenic and cell proliferation responsive to mitogen action [62]. The development of hypothalamic tissue is under control of other morphogenes, namely, bone morphogenetic proteins and fibroblast growth factors (FGF). Neurites in the hypothalamus are guided to their targets by many attractive and repulsive guidance molecules netrins, slits, semaphorins, and ephrins that have been reviewed in the context of autism elsewhere [5]. Complex molecular interactions, including the action of neuropeptide oxytocin, occur at the origin of the hypothalamic region and generation of hypothalamic cell types during development [63, 64].

## 6. Role of Hypothalamic Neuropeptides in Neurogenesis

### 6.1. Adult Hypothalamic Neurogenesis

The presence of immature mitotic neurons in the hypothalamus has been first reported by Evans et al. [65]. Recent evidence for adult hypothalamic neurogenesis has been expanded, which consequently leads to the broad discussion of details on hypothalamic neurogenic cascades, regulatory mechanisms, and potential functions [66, 67]. Adult-born neurons were found in the rat, mouse, and sheep hypothalamus [68]; however proliferating neural cells in the human hypothalamus has not yet been reliably evidenced. Hypothalamic neurogenic niche has been identified lining the ventral portion of the third ventricle [53]. Moreover, surface of the third ventricle has been suggested as a source of neurogenesis in the adult age and one study has shown that voluntary exercise correlates with proliferation of subependymal cells [2, 69]. It has been found that median eminence tanycytes (glial cells) generate newborn neurons. Tanycytes represent multipotential cells retaining the morphological features of embryonic glial cells and neural progenitor cells into adulthood [69]. Nevertheless, identity of the hypothalamic neural progenitor cells still remains controversial. It appears that they represent self-renewing cells that give rise to new tanycytes, astrocytes, and neurons [70]. Immature migrating neurons are highly present in the vicinity of the hypothalamic neurogenic niche [71]. Migrating neurons in the hypothalamus can integrate into functional circuits and modulate brain plasticity. Newly formed neurons in the hypothalamus can synthesize and release various neuropeptides [2]. There is evidence suggesting that the newly generated hypothalamic neurons may be involved in metabolism, energy balance, and body weight [72, 73].

### 6.2. Hypothalamic Neuropeptides Controlling Neurogenesis

The potential of certain neuropeptides to affect hippocampal neurogenesis has been extensively reviewed elsewhere [7]; however the involvement of neuropeptides in hypothalamic neurogenesis is less clear. The generation of new cells in the brain has been proved under influence of certain neuropeptides (Table 1). Neuropeptide oxytocin has been reported to stimulate neurogenesis; however its effect was predominately described in the adult hippocampus [47, 74]. Moreover, oxytocin may affect expression of neurotrophic factors such as brain-derived neurotrophic factor (BDNF) and nerve growth factor (NGF), which represent important regulators of neuronal function [75]. Infusion of BDNF into the lateral ventricle results in generation of new neurons in the hypothalamus of the adult rat [76]. BDNF and many other growth factors and/or neurotrophic factors such as FGF2, ciliary neurotrophic factor (CNTF), vascular endothelial growth factor (VEGF), and transforming growth factor *α* (TGF-*α*) have been shown to regulate neural stem cells and neural progenitor proliferation in the adult rodent brain [77, 78].

One study has reported that neurogenesis occurs in the adult hypothalamus, including areas containing oxytocin and vasopressin producing neurons [74]. Other authors have reported that postnatal neurogenesis occurs in the magnocellular neurons of supraoptic and paraventricular nucleus [79]. They have speculated that different time periods of formation exist for neurons that have a specific function. Moreover, it has been reported that production rate of new neurons expressing vasopressin was positively correlated with postnatal growth of the same hypothalamic region [80]. In agreement with this finding, important role of vasopressin and CRH in the regulation of hippocampal neurogenesis has been suggested [49]. Stem-like cells have been isolated from hypothalamus with the ability to generate neurons and glia producing and secreting neuropeptides including oxytocin [81].

Few studies suggest an association between eating behavior and hypothalamic neurogenesis [28]. This makes a great potential for neuropeptides involved in neurogenesis as neural progenitor cells isolated from fetal rat hypothalamus express NPY, AgRP, and POMC [82]. Peptide melanocortin, one of the POMC products, exhibits control of feeding and energy expenditure, neuroprotection, and neurogenesis through melanocortin-4 receptor subtype (MC4R) [39]. Moreover, melanocortin-induced neurogenesis triggering the Wnt and Shh signaling pathways has been demonstrated in the model of cerebral ischemia [38]. Control of food intake regulated by orexin may include effects on neurogenesis as well. Few studies have suggested that orexin-A is involved in hippocampal neurogenesis [34, 35]. Another neuropeptide, NPY, regulates the biological dynamics of neurogenic niche [83, 84] and plays a role in the modulation of adult neurogenesis [7, 85–87]. NPY directly targets certain neural stem cell subtypes (nestin- and doublecortin-positive cells), including proliferation, differentiation, migration, and functional integration of newborn neurons. Moreover, microglia and astrocytes also appear to be responsive to the peptide [43]. NPY directly interacts with another feeding-regulatory peptide ghrelin [88]. Another study has been performed by Chang et al. These authors found that prenatal nicotine exposure stimulates neurogenesis of orexigenic peptide-expressing neurons in the offspring hypothalamus [89]. Systemic ghrelin treatment stimulated neurogenesis in the adult hippocampus in mice [90]. In the hypothalamic neuronal cells, ghrelin may act as a survival factor that preserves mitochondrial integrity and inhibits apoptotic pathways during oxygen-glucose deprivation [91]. Thus, taken together oxytocin, vasopressin, NPY, and ghrelin belong to neuropeptides likely to participate in the regulation of hypothalamic neurogenesis and differentiation. Recently, it has been demonstrated that Substance P increased proliferation of neural stem/progenitor cells in the spinal cord [41]. Although no direct effect of TRH on neurogenesis is so far known, a lot of knowledge has been gained on neurogenic effects of thyroid hormones [92]. Recent study has evidenced that CRH regulates neurogenesis. The same authors have demonstrated that CRH induced proliferation and protection from apoptosis in the human neuroblastoma cells [46].

## 7. Role of Hypothalamic Neuropeptides in Neuritogenesis

### 7.1. Hypothalamic Neuritogenesis

Reviews dealing with methodological approaches related to the analysis of hypothalamic circuitry and extensive data on the development of the major axonal tracts coursing through the hypothalamus have been recently published [27, 56, 93]. Neurite outgrowth has been studied in cultures of dissociated hypothalamic cells as well [94, 95]. Sex differences in neuritogenesis in neuronal hypothalamic cultures have been also suggested [96]. The formation of projection pathways in and out of the hypothalamus is critical for a variety of neuroendocrine functions and its postnatal regulation is under control of neuropeptides (Table 1).

### 7.2. Hypothalamic Neuropeptides Controlling Food Related Circuits

Development of certain hypothalamic circuits depends on daily energy and food requirements. Moreover, age-dependent formation of intrahypothalamic axonal connection related to regulation of food intake has been extensively described [97]. Recent studies suggest that neuropeptide oxytocin is involved in energy balance control. It has been shown that hypothalamic oxytocin pathways to the brain stem contribute to the reduction of food intake [98, 99]. Oxytocin-producing cells appear early in the development of the hypothalamus [100] and their maturation, and, in particular, their ability to produce oxytocin may influence the formation of hypothalamic circuits and growth of neurites. Several studies suggest that hypothalamic neurons expressing orexigenic and anorexigenic peptides play a role in regulation of neurite growth in early developmental stage [9]. Neurons that express *α*-MSH are particularly important for regulation of hypothalamic development. It has been shown that *α*-MSH promotes neurite elongation through MC4R G-protein coupled receptor [40]. Moreover POMC neurons together with *α*-MSH producing neurons send axonal projections to the brain stem suggesting a functional role in the control of food intake [101]. Melanocortin *α*-MSH has been found to influence the differentiation of neural processes in brain neurons via increase in the levels of neurofilament proteins [102]. Reduction of food intake and body weight regulated by *α*-MSH represents a control mechanism for maintenance of energy balance. As neuropeptides represent large group of signaling molecules, they may act on the large number of receptors and share the mechanism of action on neurite extension with other neuropeptides. It has been shown that NPY promotes axonal growth and affects growth cone turning [44]. Another orexigenic peptide orexin-A has been shown to inhibit neurite retraction [36]. The same authors also observed the effect of orexin on neuronal cytoskeleton morphologic changes of actin and vimentin [103]. It has been found that neuropeptide galanin stimulates neurite outgrowth [44, 104]. Within the hypothalamus, neurons of the suprachiasmatic nucleus contain galanin and galanin mRNA distribution has been described in the arcuate and dorsomedial hypothalamic nuclei as well [105]. Axon tip accumulation of Substance P, NPY, and galanin has been observed in the model of nerve injury suggesting their role in neurite sprouting [106]. Moreover it has been found that ghrelin acts directly on hypothalamic neurons to block axon growth and reduce the overall length of axon extensions [107]. Although not directly related to the topic of the present review, it should be mentioned that recent study demonstrated that gastric peptide ghrelin mediates neural fiber growth in the arcuate nucleus of the hypothalamus during the neonatal period [107]. Development of appetite-related hypothalamic neural projections thus remains complex involving various neuropeptides originating in the central nervous system and periphery as well.

### 7.3. Hypothalamic Neuropeptides Controlling Social Behavior Related Circuits

On the basis of functional and anatomical data, comparative studies have described “social behavior network” in mammals that represents the complex neural machinery for the regulation of social behavior [108]. As components, medial amygdala, bed nucleus of the stria terminalis, lateral septum, and ventromedial and anterior hypothalamus have been included to the circuit. These areas are all reciprocally connected and express various neuropeptides and sex steroid hormone receptors as well. Many studies have examined the role of hypothalamic neuropeptides (Figure 1) in social behavior. Recent studies have shown that olfactory receptor neurons participate in polysynaptic circuits with hypothalamic subregions, involving neuropeptides urocortins in the processing of social cues [32, 109]. It is known for a long time that vasopressin and oxytocin enhance social recognition [110]. Neural mechanisms regulating social cognition and affiliative behavior always include oxytocin action [111]. Traditionally, it is believed that oxytocin and vasopressin are released within the hypothalamic and limbic areas from axons, dendrites, and cell bodies resulting in regulation of mating, reproductive, and affiliative behavior [112]. Particularly detailed review on central oxytocin pathways in the development has been recently published [113]. Embryonic hypothalamus produces immature oxytocin and cells start to generate mature (amidated) oxytocin after birth. Authors suggest that oxytocin axons grow from hypothalamus to forebrain regions and to brain stem/spinal cord after weaning [113]. Individual oxytocin neuronal projections can be found in the bed nucleus of the stria terminalis and the lateral hypothalamic area [114]. Although the bodies of oxytocin neurons are mainly restricted to the hypothalamus, oxytocin fibers are spread throughout the entire brain [111]. Oxytocin increases the firing of inhibitory hippocampal neurons [115]. Recent study has reported that oxytocin is involved in the regulation of social behavior through special cortical circuit [116]. A number of studies suggest that oxytocin modulates social perception, social cognition, and social behavior in humans. Recent reviews have been published dealing with the role of oxytocin and vasopressin in social behavior [117, 118]. Neural circuitry for social cognition depends on oxytocin and vasopressin receptor density in specific brain regions [119, 120]. Link between the individual variation in social behavior and neuropeptidergic systems including oxytocin system has been repeatedly suggested [121]. Oxytocin has sex-specific effects and it can contribute to gregariousness in both sexes in different species [122]. It can be suggested that contribution of specific peptide cell groups in the hypothalamus is important for pair bonds.

## 8. Conclusions and Perspectives

Research during recent years has shown that hypothalamic neural organization continuously changes in response to internal and external stimuli and consequently it results in production of new cells and their differentiation. In this context, it is important to understand the role of hypothalamic neuropeptides in neurogenesis and neuritogenesis. Particularly, small-size neuropeptides may play a role in neuronal proliferation and differentiation influencing growth and guidance of neurites and participating in the formation of neural circuits in early development. Neuropeptides may affect neuronal morphology, cell shape, and arborisation of dendrites as well. Hypothalamic neuropeptides may contribute to the programming of neural progenitor cells. Determination of cell fate in the view of neuropeptide production and secretion is important point for development of functional neural circuits. Although many studies mentioned in the present review show and discuss altered number of neurons and enhanced neurogenesis, conclusions should be considered carefully as functional contribution of neuropeptides is always related to concerted effects of different signaling molecules. Moreover, short-term and long-term effects of neuropeptides differ greatly and may have opposite or variable actions on brain tissue. According to the literature, many neuropeptides show trophic effects, often site and time specific. Distribution of neuropeptides and precise knowledge of their transportation from the hypothalamus to the other brain regions are therefore crucial. Further studies should focus on neuropeptide pathways and their changes during development. Conditional formation of neuronal circuits is extremely important. Effects of neuropeptides have fairly complex consequences ranging from modulation of food intake to establishment of social bonds. Recent studies have already shown the role of oxytocin and other hypothalamic neuropeptides in early development. Maintenance of balance in the orexigenic and anorexigenic hypothalamic neuropeptides is especially important in the context of maximized energy intake and mass gain during early stages of development. Nevertheless, understanding mechanisms that facilitate the formation of neural circuits mediating food intake and establishment of social bonds may help to resolve diagnosis and treatment of developmental diseases.

## Figures and Tables

**Table 1:** Effects of small-size neuropeptides on neurogenesis and neuritogenesis.

Name	Size of amino acids	Effect on neurogenesis	Effect on neuritogenesis
Orexins (orexin-A)	33	Primary hippocampal cells [34] ↑ gyrus dentatus [34, 35]	↑ primary cortical cells [36]
Melanin-concentrating hormone (MCH)	19	Unknown	↑ SH-SY5Y cells [37]
Melanocyte-stimulating hormone (*α*-MSH, *β*-MSH)	13 and 18	↑ gyrus dentatus [38, 39]	↑ dorsal root ganglia neuron culture [40]
Substance P	11	↑ spinal neural stem cells [41]	Unknown
Enkephalins (met-enkephalin)	5	↑ SH-SY5Y cells, Neuro-2A cells [42]	↑ Neuro-2A cells [42]
Neuropeptide Y (NPY)	36	↑ gyrus dentatus [43]	↑ dorsal root ganglia neuron culture [44]
Thyrotropin releasing hormone (TRH)	3	Unknown	↑ ventral spinal cord [45]
Corticotrophin-releasing hormone (CRH)	41	↑ neural stem/progenitor cells [46]	Unknown
Oxytocin	9	↑ hippocampus [47]	↑ SH-SY5Y cells [48]
Vasopressin	9	↑ gyrus dentatus [49]	Unknown

## Abbreviations

AC: Amino acid
ACTH: Adrenocorticotropic hormone
AgRP: Agouti-related peptide
BDNF: Brain-derived neurotrophic factor
CNS: Central nervous system
CNTF: Ciliary neurotrophic factor
CRH: Corticotrophin-releasing hormone
FGF: Fibroblast growth factor
IGF: Insulin-like growth factor
MCH: Melanin-concentrating hormone
MC4R: Melanocortin-4 receptor
MSH: Melanocyte-stimulating hormone
NGF: Nerve growth factor
NPY: Neuropeptide Y
POMC: Proopiomelanocortin
Shh: Sonic hedgehog protein
TGF-*α*: Transforming growth factor *α*
TRH: Thyrotropin releasing hormone
VEGF: Vascular endothelial growth factor
Wnt: Wingless family proteins.
